# S100 Protein and Interleukin Biomarkers Among COVID-19 Subjects With and Without Pneumonia: A Systematic Review and Meta-Analysis

**DOI:** 10.3389/bjbs.2025.15355

**Published:** 2026-01-08

**Authors:** Haily Liduin Koyou, Vasudevan Ramachandran, Mohd Nazil Salleh, Wan Aliaa Wan Sulaiman, Mohd Hazmi Mohamed, Mohd Jaamia Qaadir Mohd Badrin, Caroline Satu Jelemie

**Affiliations:** 1 Department of Medical Sciences, Faculty of Health Sciences, University College of MAIWP International, Kuala Lumpur, Malaysia; 2 RADILAB Diagnostics Sdn. Bhd., Kuala Lumpur, Malaysia; 3 Department of Conservative Dentistry and Endodontics, Saveetha Institute of Medical and Technical Sciences, Saveetha Dental College and Hospitals, Saveetha University, Chennai, India; 4 Malaysian Research Institute on Ageing, Universiti Putra Malaysia, Serdang, Selangor, Malaysia; 5 Department of Medicine, Faculty of Medicine and Health Sciences, Universiti Putra Malaysia, Serdang, Selangor, Malaysia; 6 Department of Otorhinolaryngology, Head and Neck Surgery, Faculty of Medicine and Health Sciences, Universiti Putra Malaysia, Serdang, Selangor, Malaysia; 7 Department of Health Diagnostics, Faculty of Health Sciences, Universiti Selangor, Shah Alam, Malaysia; 8 Department of Nursing, Faculty of Medicine and Health Sciences, Universiti Malaysia Sabah, Kota Kinabalu, Sabah, Malaysia

**Keywords:** COVID-19, biomarkers, pneumonia, interleukin, S100 proteins

## Abstract

**Background:**

The global spread of COVID-19, caused by SARS-CoV-2, has resulted in a wide spectrum of clinical manifestations, ranging from asymptomatic cases to severe complications, such as pneumonia, acute respiratory distress syndrome (ARDS), and multiple organ failure. Identifying effective biomarkers is essential for predicting disease severity and improving patient management.

**Objectives:**

This meta-analysis aims to assess the significance of S100 proteins (S100A4, S100A8, S100A9, S100A12, S100B, S100P) and interleukins (IL) (IL-6, IL-8, IL-10, IL-17, IL-1β) in COVID-19 patients, comparing those with and without pneumonia or organ failure.

**Methods:**

A systematic literature search was conducted on different databases, yielding 47 relevant studies published between 2020 and 2024. Data on the prevalence of IL and S100 protein levels were extracted and analyzed using pooled standardized mean differences (SMD) and heterogeneity (I^2^) to evaluate their associations with disease severity.

**Results:**

IL-6 and IL-10 levels were significantly elevated in COVID-19 patients suffering from pneumonia or organ failure. IL-6 levels were notably higher in pneumonia patients compared to those without (SMD = 0.34 [95% CI: 0.17, 0.52], I^2^ = 29%). Similarly, elevated S100B levels were observed in severe cases (SMD = 0.51 [95% CI: 0.19, 0.83], I^2^ = 0%). While IL-10 levels showed high variability (I^2^ = 90%), they remained consistently linked with worse outcomes.

**Conclusion:**

This meta-analysis underscores the potential of IL-6, IL-10, and S100 proteins as important biomarkers in evaluating COVID-19 severity, offering valuable insights to help clinical management.

## Introduction

The COVID-19 pandemic, caused by the SARS-CoV-2 virus, has led to substantial global morbidity and mortality, with more than 770 million confirmed cases and nearly 7 million deaths reported cumulatively as of 2024, averaging over 150 million new cases and more than 1 million deaths per year since 2020 [1]. In addition to this human toll, the pandemic has placed an unprecedented economic burden on healthcare systems, costing billions of dollars and creating long-term structural challenges [2]. Clinically, COVID-19 presents across a broad spectrum, ranging from asymptomatic or mild infection to severe disease marked by pneumonia, acute respiratory distress syndrome, multi-organ failure, and death [1]. This variability in outcomes underscores the urgent need for reliable biomarkers to predict disease severity and guide clinical management.

In this context, biomarkers related to inflammatory responses, particularly the S100 protein family and various interleukins (IL), have drawn considerable attention for their potential roles in the pathogenesis of COVID-19. The S100 protein family, which includes members such as S100A4, S100A8, S100A9, S100A12, S100B, and S100P, is known to participate in inflammatory processes and has been implicated in various disease states, including respiratory infections [3]. Concurrently, interleukins like IL-6, IL-8, IL-10, IL-17, and IL-1β are vital mediators of inflammation and have been shown to correlate with the severity of the disease in COVID-19 cases [4, 5].

A key aspect of severe COVID-19 is the overactive host-defence response, often called “cytokine storm,” Where cytokines that promote inflammation, like IL-6 and IL-10, significantly influence disease progression [6]. Raised IL-6 concentrations have been consistently associated with severe respiratory complications and for intensive care. IL-10, typically regarded as an anti-inflammatory cytokine, also shows increased levels in severe cases, indicative of systemic immune dysregulation [4]. These cytokines are integral to the hyperinflammatory response leading to tissue damage and organ dysfunction in affected patients [7].

In addition to cytokines, the S100 protein family is emerging as a group of biomarkers of interest in the realm of COVID-19 research [8]. S100B, commonly associated with neuroinflammatory processes, is released during systemic inflammatory reactions and may serve as an indicator of endothelial injury and cellular damage in patients experiencing severe forms of the disease [9]. Additionally, calprotectin (S100A8/A9) has been shown to rise in patients with significant inflammatory conditions, potentially reflecting the inflammatory state of COVID-19 patients [10].

Emerging evidence underscores that heightened values of IL-6 with other inflammatory cytokines closely correspond to adverse outcomes in COVID-19 [4, 11]. Furthermore, research has suggested that specific metabolic profiles characterized by certain biomarkers may predict the likelihood of developing intense pneumonia in COVID-19 patients [12]. A thorough comprehension of the prevalence and implications of S100 proteins and interleukins in the context of COVID-19 it plays a key role in formulating specific treatment strategies and enhancing patient management.

While prior meta-analyses and systematic reviews have primarily focused on the role of cytokines or individual interleukins, such as IL-6, in predicting COVID-19 severity, there is limited consolidated evidence on both S100 proteins and interleukins as a combined biomarker profile. This meta-analysis addresses this gap by synthesizing data on S100 proteins and interleukin markers to provide a multi-marker perspective on their association with disease severity in COVID-19 patients.

This meta-analysis aims to consolidate existing research on S100 proteins and interleukin markers in individuals diagnosed with COVID-19. Specifically, the analysis has three primary objectives: first, to determine the frequency of S100 protein family biomarkers in patients with and without pneumonia or organ failure (defined as the loss of function in a vital organ, such as the lungs, heart, kidneys, or liver); second, to evaluate the prevalence of interleukin markers in comparable cohorts; and third, to synthesize current literature to deepen understanding of the relevance of these biomarkers in the context of disease severity. The present systematic review also endeavors to shed light on the association within these biomarkers and disease severity, contributing to the broader discourse on the inflammatory mechanisms underlying the pathology of COVID-19. Insights derived from this synthesis will be instrumental in guiding clinical practice and informing future research directions as the pandemic continues to evolve.

## Materials and Methods

### Search Strategy

**FIGURE 1:**
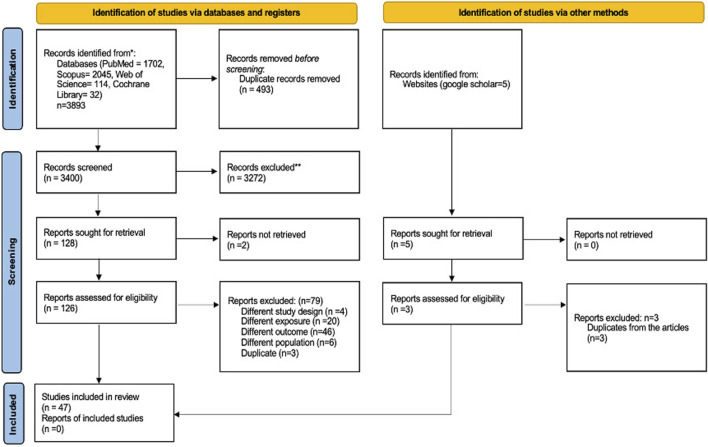
Flow diagram showing the study selection stages according to PRISMA.

A comprehensive search approach was crafted to identify relevant articles across major databases, specifically Web of Science, PubMed, Scopus, Web of Science, and the Cochrane Library, from January 2020 to September 2024. Appendix 1 provides a detailed description of this strategy. Additionally, Google Scholar was reviewed to ensure any additional pertinent studies were captured. The search terms were thoughtfully identified and crafted using a composite of the following keywords: “COVID-19,” “S100 Proteins,” “Pneumonia,” and “Interleukins.” The methodology for this systematic review adhered to the “Preferred Reporting Items for Systematic Reviews and Meta-Analyses (PRISMA)” guidelines (as illustrated in Figure 1).

### Eligibility Criteria

The review included studies investigating the expression of biomarkers, specifically S100 proteins and interleukin markers, in COVID-19 patients, with and without pneumonia or organ failure. The selected articles comprised a range of study designs, including interventional trials and observational studies, such as prospective and retrospective cohort studies, nested studies, case-control studies (both matched and unmatched), cross-sectional analyses, and case reports. Exclusion criteria encompassed publications of non-pertinent formats (e.g., letters to the editor, editorials, or comments), non-English articles, and studies whose data did not align with the primary focus of the review.

### Study Selection Process

The study screening process was completed in two phases. Firstly, two reviewers (VD and WAWS) independently reviewed the titles and abstracts of the retrieved studies to identify those meeting the inclusion criteria. In the second phase, the same reviewers examined the full texts for potentially relevant studies, including only those that aligned with the criteria. Any disagreements between researchers were resolved through discussion and consensus, with a third reviewer (MNS) stepping in for arbitration. For this meta-analysis, disease severity was operationalised according to the categories reported in the included studies. Specifically, we compared biomarker profiles between (i) COVID-19 patients with pneumonia and (ii) COVID-19 patients without pneumonia. In addition, where studies reported data, we included analyses of (iii) COVID-19 patients with organ failure versus those without organ failure. Several studies also provided data on (iv) healthy individuals, who were included as additional comparator groups to provide context for interpreting biomarker elevations.

### Data Extraction Process

An MS Excel-based data extraction form was developed following detailed discussions with the review team to ensure consistency. The form captured comprehensive information, organized into key categories: study identifiers (“author, study year, publication year”), article origin details (“country, study setting, publication type”), and study specifics (“design, patient demographics, baseline characteristics, and main findings”). Two researchers independently conducted data extraction, and any discrepancies or inconsistencies were resolved through discussion, consensus-building, or by involving a third team member.

### Risk of Bias Assessment

The Newcastle-Ottawa Scale (NOS), a standard tool for observational studies, was used to assess the quality of the included studies. The NOS was applied separately to cohort and case-control studies, with an adapted version used for cross-sectional studies. This scale assigns up to 9 points across three domains: “selection,” “comparability,” and “outcome or exposure assessment.” Studies were classified based on their scores: low quality (0–3 points), moderate quality (4–6 points), and high quality (7–9 points).

### Synthesis Methods

A meta-analysis was conducted to estimate the prevalence of COVID-19, with or without associated pneumonia or organ failure, in relation to S100 proteins and interleukin markers. Forest plots were generated to visually represent the collated data and the effect estimates across studies. Heterogeneity was evaluated using the χ^2^ test with a significance level of 5%, and effect sizes were expressed as standardized mean differences (SMDs). The degree of heterogeneity was quantified using the I^2^ statistic, with values above 50% signifying moderate heterogeneity among the included studies. All analyses were performed using RevMan software. Patients were stratified by disease severity into subgroups, specifically COVID-19 with or without pneumonia or organ failure, while several studies also included healthy controls as comparator groups. This classification enabled consistent subgroup analyses; however, it should be acknowledged that the included studies did not uniformly apply standardized severity definitions, such as those proposed by the World Health Organization (WHO) or the National Institutes of Health (NIH).

## Results

The initial search yielded 3,893 records relevant to the study objectives. After applying the predefined inclusion criteria, 128 records proceeded to the title and abstract screening phase and the screening led to the exclusion of 79 records. 47 studies were deemed eligible for data extraction and are presented in Figure 1 and Table 1. No additional study was identified through the Google Scholar database.

**TABLE 1:** Overview of included study characteristics.

No	Author	Country	Year	Duration	Settings	Study design	Sample size	Age (years)	Sex, M/F	Diabetes	Hypertension
1	Yueting Tang [13]	China	2021	20 January to 27 February 2020,	Hospitalised patients	Cohort	71	60 (47–68) median (IQR)	Male: 27 (38.0) Female: 44 (62.0)	10 (14.1)	23 (32.4)
2	Sanaz Paikar [14]	Iran	2024	-	Hospitalised patients	Cohort	120	-	-	-	-
3	Robert Chrzan [15]	Poland	2021	20 January 2021 to 31 May 2021	Hospitalised patients	Cross sectional	388	60.5 (mean)	146 women, 242 men	-	-
4	Wandong Hong [16]	China	2020	January 2020 and March 2020	Hospitalised patients	Cross sectional	63	56 ± 15 mean ± SD	41 (65.1) (n%) male	-	-
5	Juan Sebastián Henao-Agudelo [17]	Colombia	2024	January and June 2021	Hospitalised patients	Cohort	40	43 ± 11.2 mean ± SD	Male: 28/40 (70)	1 (2.5)	3 (7.5)
6	Guang Chen [18]	China	2020	Late December 2019 to 27 January 2020	Hospitalised patients	Cross sectional	21	56.0 (50.0–65.0) median (IQR)	Male: 17 (81.0%)	3 (14.3)	5 (23.8)
7	Hitoshi Kawasuji [19]	Japan	2022	January 2022 and March 2022	Hospitalised patients	Cohort	67	62 (49–73)	Male: 46 (68.7%)	11 (16.4)	29 (43.3)
8	Kentaro Nagaoka [20]	Japan	2023	December 2020 and April 2022	Hospitalised patients	Cohort	187	-	-	​	​
9	Maimun Z Arthamin [21]	Indonesia	2023	18 April and 28 June 2021	Hospitalised patients	Cross sectional	81	-	-	28.39%	18.52%
10	You Xu [22]	China	2023	December 2022 to January 2023	Hospitalised patients	Cross sectional	109	-	-	-	-
11	Muhammet Yusuf Tepebaşı [23]	Turkey	2022	-	Hospitalised patients	Cross sectional	95	-	-	-	-
12	Diana Fuzio [24]	Italy	2021	March to June 2021	Hospitalised patients	Cross sectional	150	70 (62–80) median (IQR)	Male −79 (52.7%)	-	-
13	Felix Eduardo R. Punzalan [25]	Philippines	2021	October 2020 to September 2021	Hospitalised patients	Cohort	400	-	-	-	-
14	C. H. Krishna Reddy [26]	India	2022	June 2020 to May 2021	Hospitalised patients	Cross sectional	210	-	-	-	-
15	Kentaro Nagaoka [27]	Japan	2022	April 2021 and June 2021	Hospitalised patients	Cohort	50	50 (34–57) median (IQR)	33/17 (m/f)	5 (10)	12 (24)
16	Federica Tonon [28]	Italy	2022	October 2020 and April 202	Hospitalised patients	Case-control	84	64	Male: (71%)	​	​
17	Pedro Martínez-Fleta [29]	Spain	2021	10th March to 21st April 2020	Hospitalised patients	Cohort	85	64 (55–76) median (IQR)	Male: 35 (41.18)	19 (22.35)	36 (42.35)
18	Wendy Fonseca [30]	USA	2021	March 2020 to September 2020	Biobank	Cohort	56	-	-	-	-
19	Miao Wang [31]	China	2020	10 February 2020 to 30 March 2020	Hospitalised patients	Cross sectional	77	-	-	-	-
20	Li-Da Chen [32]	China	2020	28 January 2020 and 30 March 2020	Hospitalised patients	Cross sectional	96	52.75 ± 16.09 mean ± SD	Male: 53 (50.0)	13 (12.3)	17 (16.0)
21	Shirin Assar [33]	Iran	2023	-	Hospitalised patients	Cohort	60	​	​	​	​
22	Abdurrahim Kocyigit [34]	Turkey	2021	June 2020 and July 2020	Hospitalised patients	Case-control	52	-	-	-	-
23	Archana Kulkarni‐Munje [35]	India	2021	20th April 2020 to 11th June 2020	Hospitalised patients	Cohort	70	-	-	-	-
24	Unzela Ghulam [36]	Pakistan	2022	​	Hospitalised patients	Cross sectional	118	-	Male: 76 (64.4%)	​	​
25	Mingming Jin [37]	China	2020	27 January 2020 to 9 March 2020	Hospitalised patients	Cross sectional	311	-	-	-	-
26	Marco Contoli [38]	Italy	2021	1 April until the end of May 2020	Hospitalised patients	Cohort	65	-	-	-	-
27	Agnes S. Meidert [39]	Germany	2021	03/2020 and 04/2020	Hospitalised patients	Cohort	100	-	-	-	-
28	Parvez Anwar Khan [40]	India	2022	1 March 2020 and 8 April 2020,	Hospitalised patients	Cross sectional	83	-	-	-	-
29	Fatma Kesmez Can [41]	Turkey	2021	-	Hospitalised patients	Cohort	90	-	-	-	-
30	Jose J. Guirao [42]	Spain	2020	1 April 2020 and 30 April 2020	Hospitalised patients	Cohort	50	-	-	-	-
31	Peng-Hui Yang [43]	China	2020	27 December 2019 to 12 March 2020	Hospitalised patients	Cohort	70	45.00 (34.50–61.00) median (IQR)	Male: 38 (54.3), female: 32 (45.7)	7 (10.0)	17 (24.3)
32	S. Keddie [44]	UK	2020	-	Hospitalised patients	Cohort	100	59 (20–92) median (IQR)	Male: 74 (75%)	25	37
33	Florian Brandes [45]	Germany	2021	-	Medical centres	Cohort	577	-	-	-	-
34	Aishwarya K Marimuthu [46]	India	2021	May 2020 to July 2020	Hospitalised patients	Cross sectional	221	60 (mean)	Male: 70.1% and female: 29.9%	111	101
35	Masaya Sugiyama [47]	Japan	2020	January to May 2020	Hospitalised patients	Cohort	28	-	-	-	-
36	Y Z Zhou [48]	China	2020	December 2019 and February 2020	Hospitalised patients	Cohort	140	-	-	-	-
37	Francesco Taus [49]	Italy	2020	March 25 and 3 May 2020	Hospitalised patients	Cohort	65	61.8 ± 13.4 (47–94) mean ± SD (range)	Male: 18 (49%) Female: 19 (51%)	​	21/37 (56.8%)
38	Fang Liu [50]	China	2020	18 January 2020, and 12 March 2020	Hospitalised patients	Cross sectional	140	65.5 (54.3–73.0) median (IQR)	Female: 91 (65.0%) male: 49 (35.0%)	34 (24.3%)	63 (45%)
39	Huan Han [51]	China	2020	Jan 2020 and February 2020	Hospitalised patients	Cross sectional	102	-	-	-	-
40	Maximilian Robert Gysan [52]	Austria	2023	06.01.2021 and 31.05.202	Hospitalised patients	Cohort	88	68 (55–77) median (IQR)	Male: 61 (69.1%)	22 (25%)	41 (46.6%)
41	Olga Kalinina [53]	Russia	2022	-	Hospitalised patients	Cohort	84	-	-	-	-
42	Chunjin Ke [54]	China	2020	-	Hospitalised patients	-	194	64 (54–71.25) median (IQR)	Male: 115 (59.27)	39 (20.10)	73 (37.63)
43	Nikolaos K. Gatselis [55]	Greece	2023	March 2020 to August 2021	Hospitalized due to COVID-19 related pneumonia	Cohort	736	63 (22) median (IQR)	Male: 428 (58.2)	-	-
44	Ergun Mete [56]	Turkey	2021	26 January −10 March 2021	Hospitalized: Outpatient clinic of our ED	Case control	64-Cases, 30 control	-	-	-	-
45	Ryan C. Silva [57]	Brazil	2023	2nd semester of 2020 to the 1st semester of 2021	Hospitalised patients	Case control	141	43 ± 13 mean ± SD	Female: 62 (44%) male: 79 (56%)	-	-
46	Antonio Aceti [58]	Italy	2020	29th January to 6th May 2020	Hospitalised patients	Cohort	74	66 (32–89) median (IQR)	Female: 25 (49%)	-	-
47	Tezcan Kaya [59]	Turkey	2021	1 November 2020 and 10 December 2020	Hospitalised patients	Cross sectional cohort	​	66.5 ± 15.7 mean ± SD	-	-	-

Age values are reported exactly as in the original studies: mean ± SD, median (IQR), or median (range). “–” indicates Not Reported (NR). In some studies, only the IQR range was given.

The included studies, published between 2020 and 2024, comprised predominantly cohort designs (n = 27), followed by cross-sectional (n = 16) and case–control studies (n = 4). Sample sizes ranged from 21 to over 500 hospitalized COVID-19 patients, spanning diverse geographic regions such as India, the United Kingdom, and Italy, with the majority conducted in China. Age was reported in 28 of the 47 studies, ranging from 20 to 94 years, with most cohorts clustering between 55 and 65 years. Reporting formats varied across studies, including mean ± SD, median with interquartile range (IQR), or median with range, while several studies did not report age or provided only IQR width. Sex distribution was available in most, though not all, studies, with male representation ranging from 35% to 81% and female representation from 19% to 65%. Only a small number of studies reported age stratified by sex, limiting more granular demographic analyses.

Across the included studies, significant focus has been placed on cytokine levels, particularly IL-6 and IL-10, as key markers associated with disease severity and progression. In studies from China, Poland, and Colombia, patients with severe and critical COVID-19 cases were found to have elevated levels of these markers compared to moderate or mild cases. Notably, IL-6 values were consistently increased in patients accompanied by pneumonia or requiring advanced respiratory support, underscoring its importance as a key indicator of inflammation and clinical burden. Several studies also included healthy individuals (donors/volunteers) as controls to facilitate comparison. Comorbidity data for healthy controls were generally not reported in the included studies.

Elevated IL-2R, IL-8, and IL-18 levels were observed in patients with poor clinical outcomes, particularly those with severe respiratory symptoms and organ failure. In our pooled analyses, IL-6 was significantly higher in COVID-19 patients with pneumonia compared to those without pneumonia (SMD = 0.34, 95% CI 0.17–0.52, p < 0.0001), and also higher in COVID-19 pneumonia cases compared to healthy controls (SMD = 0.49, 95% CI 0.31–0.68, p < 0.00001). IL-8 was also elevated in COVID-19 pneumonia versus healthy controls (SMD = 0.59, 95% CI 0.20–0.98, p = 0.003). IL-10 was strongly elevated in COVID-19 pneumonia versus healthy controls (SMD = 1.26, 95% CI 0.96–1.57, p < 0.00001), but the comparison between COVID-19 with pneumonia and COVID-19 without pneumonia groups was not significant (SMD = 0.15, 95% CI –0.24–0.54, p = 0.45). These results highlight IL-6 and IL-10 as potential early indicators of disease progression, while also underscoring heterogeneity in IL-10 findings across study designs. Several studies reported a higher prevalence of diabetes and hypertension in patients with severe COVID-19, where cytokine levels were also elevated. On the other hand, serum S100B levels were markedly higher in both early- and late-stage COVID-19 patients compared with healthy individuals. Elevated S100B levels were linked to worse clinical status, with significantly higher levels observed in COVID-19 patients who required intensive care unit (ICU) oxygenation compared with those managed without ICU support.

### Meta-Analysis

Each figure represents an independent subgroup comparison—pneumonia versus healthy controls, and pneumonia versus non-pneumonia COVID-19 cases, respectively. These analyses are presented separately and are not intended for direct cross-comparison.

#### IL-6, IL 8 and IL10 Markers

**FIGURE 2:**
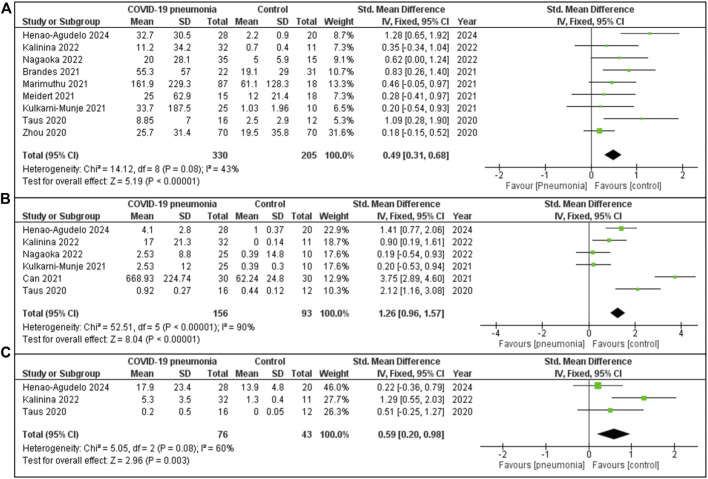
**(A)** IL-6 expression in COVID-19 patients with pneumonia vs. Healthy individuals. **(B)** IL-10 concentration in COVID-19 cases with pneumonia vs. Healthy controls group. **(C)** IL-8 quantity in COVID-19 cases with pneumonia vs. Healthy controls.

The meta-analysis demonstrates a consistent trend of significantly elevated IL-6 levels in patients with COVID-19 pneumonia compared to healthy controls (Figure 2A). The aggregate effect size is moderate (SMD = 0.49 indicating a notable difference in IL-6 concentrations between the groups. This analysis incorporates data from nine studies, each contributing variable statistical weight: the largest contribution came from Zhou (31.6%) [47] and the smallest from Taus [48] (5.3%). Although there was some variability among studies (I^2^ = 43%), the pooled effect reveals a statistically significant difference between COVID-19 pneumonia patients and the healthy individuals. The studies analysed included 330 patients in the COVID-19 pneumonia cohort and 205 individuals in the healthy or non-COVID-19 cohort.

The Figure 2B meta-analysis consists of six studies, with the largest contribution from Henao Agudelo 2021 and the smallest from Taus [48], reflecting varying study weights. The overall pooled SMD was 1.26 [95% CI: 0.96, 1.57], indicating that COVID-19 pneumonia patients, on average, exhibit significantly higher values for the measured IL 10. Although this difference was significant, substantial heterogeneity exists across the studies (I^2^ = 90%). The overall effect was statistically significant (Z = 8.04, P < 0.00001).

The meta-analysis (Figure 2C) evaluates levels of IL-8 in COVID-19 pneumonia individuals compared to a healthy cohort, based on three included studies. The largest contribution was from Henao-Agudelo 2024 (46.0%), followed by Kalinina [53] (27.7%) and Taus [48] (26.3%). The pooled SMD is 0.59 [95% CI: 0.20, 0.98], indicating that IL-8 levels were moderately higher in COVID-19 pneumonia patients compared to the control group. The studies included 76 patients within the COVID-19 pneumonia group and 43 individuals in the healthy or non-COVID-19 control group. Moderate heterogeneity was observed (I^2^ = 60%, P = 0.08). The overall effect is statistically significant (Z = 2.96, P = 0.003), reflecting a marked difference in IL-8 levels between COVID-19 pneumonia patients and the healthy controls.

#### S100B Markers

**FIGURE 3:**
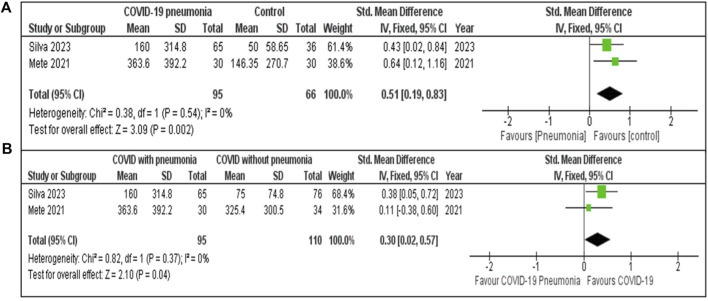
**(A)** S100B concentration in COVID-19 pneumonia cases compared to Healthy controls. **(B)** S100B concentration in COVID-19 patients with and without pneumonia.

The Figure 3A meta-analysis presented evaluates levels of S100B based on two studies. The pooled SMD is 0.51 [95% CI: 0.19, 0.83], indicating that S100B levels were moderately higher in COVID-19 pneumonia patients compared to the healthy controls. The heterogeneity was low (I^2^ = 0%, P = 0.54), suggesting minimal variability between the studies. The significant effect (Z = 3.09, P = 0.002) reveals a clear difference in S100B levels between patients with COVID-19 pneumonia and healthy controls.

The meta-analysis (Figure 3B) included two studies. The pooled SMD for S100B COVID-19 with and without pneumonia was 0.30 [95% CI: 0.02, 0.57], indicating that S100B levels were moderately higher in COVID-19 pneumonia patients compared to those without pneumonia. Low heterogeneity (I^2^ = 0%, P = 0.37) with a statistically significant effect (Z = 2.10, P = 0.04).

#### COVID-19 With and Without Pneumonia

**FIGURE 4:**
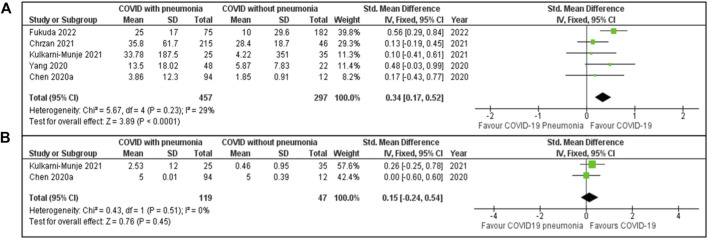
**(A)** IL-6 concentration among COVID-19 patients with and without pneumonia. **(B)** IL-10 values in COVID-19 patients with and without pneumonia.

Figure 4A presents findings based on data from five studies. These studies included 457 patients in the COVID-19 pneumonia group and 297 patients without pneumonia. The combined SMD was 0.34 [95% CI: 0.17, 0.52]. Low heterogeneity was observed (I^2^ = 29%, P = 0.23). The overall effect (Z = 3.89, P < 0.0001), signifies a clear difference in IL-6 levels between the groups.

The forest plot Figure 4B examines IL-10 levels using data from two studies. The meta-analysis includes two studies, with Chen [18] contributing 42.4% of the weight and Kulkarni-Munje [34] contributing 57.6%. The pooled SMD is 0.15 [95% CI: −0.24, 0.54]. The heterogeneity is low (I^2^ = 0%, P = 0.51), suggesting consistency between the studies. The aggregated effect was not statistically significant (Z = 0.76, P = 0.45).

#### COVID-19 With and Without Organ Failure

**FIGURE 5:**
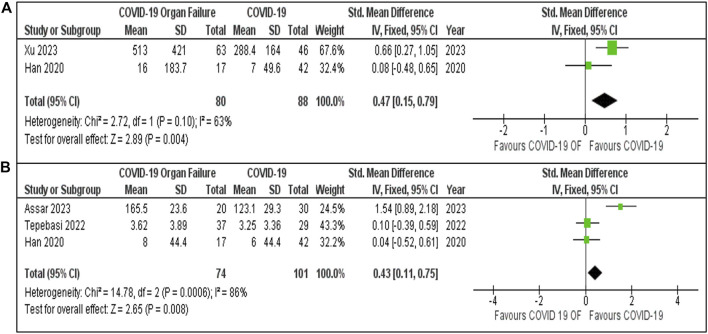
**(A)** IL-6 Levels in COVID-19 patients with and without Organ Failure. **(B)** IL-10 Levels in COVID-19 patients with and without organ failure.

Meta-analyses of IL-6 and IL-10 levels in COVID-19 patients with and without organ failure (Figures 5A,B) provide insights into the differences between these two groups. For IL-6, two studies—Xu [22] (67.6%) and Han [50] (32.4%)—were included, with a pooled of 0.47 [95% CI: 0.15, 0.79]. Study heterogeneity was moderate (I^2^ = 63%, P = 0.10). For IL-10 levels, three studies were analysed, yielding a pooled SMD of 0.43 [95% CI: 0.11, 0.75]. In both analyses, COVID-19 patients with organ failure showed significantly higher IL-6 and IL-10 levels than those without organ failure, with greater variability in IL-10 findings (I^2^ = 86%, P = 0.0006).

### Quality Assessment

**FIGURE 6:**
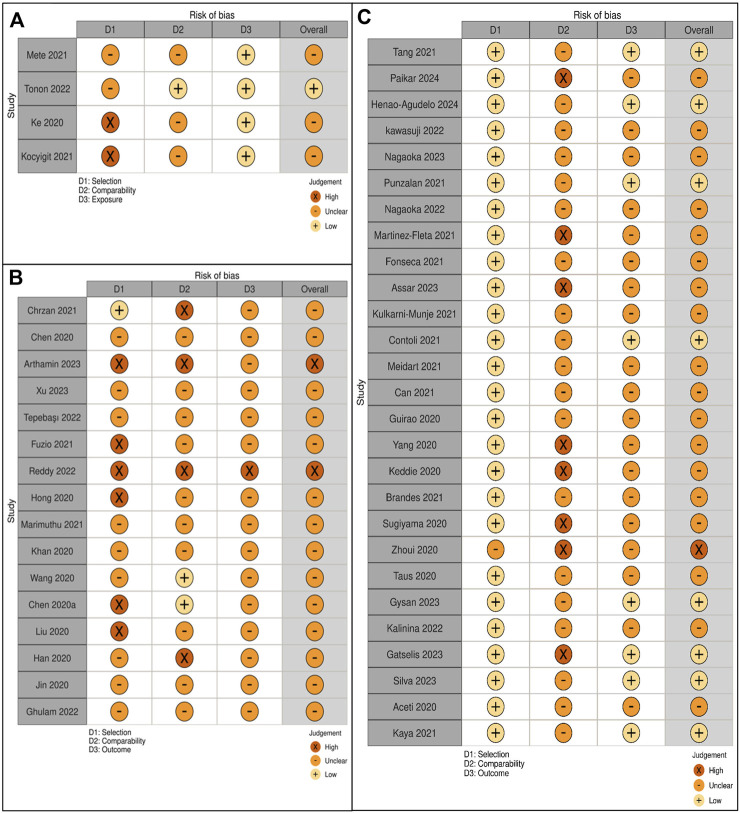
**(A)** Quality assessment of case-control studies. **(B)** Quality assessment of cross-sectional studies. **(C)** Quality assessment of cohort studies.

The NOS was adapted for cohort, case-control, and cross-sectional studies to evaluate study quality (Figures 6A–C). Out of 51 studies, 14 (27%) had low risk, 35 (69%) moderate risk, and 2 (4%) high risk of bias, with quality scores ranging from 3 to 8 points.

## Discussion

This meta-analysis synthesizes studies from 2020 to 2024 on S100 proteins and interleukin markers in COVID-19, with a primary focus on pneumonia versus non-pneumonia cases. In studies that included healthy controls, these data provided additional insight, highlighting the extent of biomarker elevations in COVID-19 and reinforcing their relevance to disease severity. The findings reinforce the importance of these biomarkers in elucidating the inflammatory pathways involved in COVID-19, while also suggesting their potential for clinical application in predicting disease trajectory and outcomes. Elevated levels of pro-inflammatory cytokines, such as IL-6, IL-1β, and TNF-α, are consistently associated with disease progression and poor prognosis in COVID-19 patients [13, 14]. Cytokine profiles, including elevated IP-10, MIP1α, and IL-6, have been linked to severe disease [14], while immune responses, such as reduced lymphocyte counts and increased CD8^+^ T cell exhaustion, correlate with adverse outcomes [22, 42]. Studies have also highlighted the role of biomarkers like S100B and calprotectin in assessing disease severity and predicting neurological and systemic complications [56, 57, 60, 61]. Advanced diagnostic tools, including AI-guided high-resolution computed tomography (HRCT) assessments, have been developed to predict pneumonia severity by analyzing clinical and immune parameters [15]. These markers, alongside others like CCL17, CRP, and procalcitonin, have shown potential in distinguishing between mild and severe disease [39, 46]. Furthermore, the adiponectin-to-leptin ratio has emerged as a promising predictor of pneumonia severity [28].

### Interleukin Markers in Severe COVID-19

A major observation in this meta-analysis is the significant relationship between elevated interleukin levels, mainly IL-6 and IL-10, and adverse COVID-19 outcomes. IL-6 is a well-established pro-inflammatory cytokine that contributes to the inflammatory cascade, a hallmark of acute COVID-19 patients, particularly in patients requiring advanced respiratory support or critical care. Consistent with earlier studies, such as the RECOVERY trial [62, 63], our results demonstrate that IL-6 levels are significantly higher in COVID-19 patients with pneumonia compared with those without pneumonia, and also compared with healthy controls, confirming its role in exacerbating hyperinflammatory responses.

Despite its anti-inflammatory nature, IL-10 is also markedly increased in acute COVID-19 patients, reflecting a broader dysregulation of the immune system [64]. Our findings revealed a notable elevation of IL-10 in COVID-19 patients who developed organ failure compared with those without organ failure, consistent with previous literature that highlights IL-10’s role in severe disease progression [11]. While IL-6 is widely recognized as a driver of pro-inflammatory activity, the rise in IL-10 levels could signify the body’s attempt to regulate the excessive inflammation; although, it appears insufficient to counterbalance the immune dysfunction. The observed heterogeneity in IL-10 levels across different studies highlights the complexity of immune responses in COVID-19, indicating that IL-10 may be more indicative of generalized immune dysregulation than inflammation alone. This aligns with other studies indicating that immune cell subsets, particularly CD8^+^ T cells, play a crucial role in COVID-19 outcomes, with altered lymphocyte counts correlating to worse prognosis [22].

### S100 Proteins as Biomarkers of Inflammation

This meta-analysis also highlights the involvement of S100 proteins, particularly S100B and calprotectin (S100A8/A9), in COVID-19 pathophysiology. Both markers are well known for their roles in modulating immune and inflammatory responses, and their elevated values in COVID-19 cases were consistently observed in patients with more severe disease presentations, supporting their potential role as indicators of severity [65]. S100B, a protein typically linked to neuroinflammation, was found to be significantly elevated in COVID-19 pneumonia patients compared with healthy controls (SMD = 0.51, 95% CI 0.19–0.83, p = 0.002). When comparing COVID-19 patients with and without pneumonia, S100B was also higher in the pneumonia group (SMD = 0.30, 95% CI 0.02–0.57, p = 0.04). These findings suggest that S100B is not only elevated in COVID-19 but may also reflect greater inflammatory burden in patients who progress to pneumonia [56]. Although the included studies largely reported biomarkers independently, the strength of this meta-analysis lies in synthesizing IL-6, IL-10, IL-8, and S100B together. By evaluating these markers side by side, our findings provide a broader multi-marker perspective that reflects different aspects of the inflammatory and tissue injury response in COVID-19, and offers a basis for future studies to assess their combined predictive value.

Calprotectin (S100A8/A9), recognized for its role in neutrophil activation and systemic inflammation, also showed elevated levels in patients requiring intensive care. This finding corroborates earlier studies, which have demonstrated that higher calprotectin levels are predictive of poor outcomes in respiratory infections [55]. The elevated levels of S100A8/A9 in COVID-19 patients, particularly those with severe pneumonia and multi-organ failure, suggest its potential as a biomarker for disease progression, helping to predict which patients may require intensive care or more aggressive treatment [66]. This aligns with studies that demonstrate the relevance of these biomarkers in predicting complications like thromboinflammation and respiratory failure [25, 41].

### Comparative Findings and Study Heterogeneity

The variability in findings related to IL-6 and IL-10, particularly in patients with organ failure, indicates differences in cytokine expression in relation to clinical severity. Such variability is understandable, given the diversity of patient populations, underlying health conditions, and treatment regimens included in the analysis. Nonetheless, it underscores the need for more consistent methodologies for assessing cytokine levels in clinical practice. For example, the substantial heterogeneity (I^2^ = 90%) seen in IL-10 studies may reflect differences in patient demographics and the timing of cytokine measurement, which could contribute to the differing outcomes observed [6, 11].

In contrast, the low heterogeneity (I^2^ < 30%) across studies examining IL-6 and S100B levels in patients with pneumonia indicates more uniform findings [67, 68]. This consistency strengthens the case for using these markers as reliable predictors of disease severity, particularly in patients presenting with COVID-19 pneumonia. The low variability suggests that these biomarkers may have greater utility in clinical settings for the early identification of high-risk patients. Additionally, biomarkers such as CCL17, CRP, and procalcitonin, along with the adiponectin-to-leptin ratio, have shown promise in distinguishing between mild and severe disease, further supporting their use in clinical practice [28, 46]. To avoid misinterpretation, it should be emphasized that analyses comparing COVID-19 pneumonia with healthy controls and those comparing pneumonia with non-pneumonia COVID-19 reflect different cohorts and therefore cannot be directly compared.

### Clinical Relevance and Future Research

The findings of this meta-analysis have important implications for clinical practice. The consistent association of elevated IL-6, IL-10, and S100B levels with severe disease outcomes points to their potential use in stratifying COVID-19 patients based on risk [69]. Early identification of patients with heightened levels of these biomarkers could facilitate timely intervention with anti-inflammatory treatments or more intensive monitoring. For example, elevated IL-6 has been incorporated into risk stratification protocols to identify patients who may benefit from early administration of immunomodulatory therapies such as tocilizumab, with several studies reporting reduced progression to respiratory failure and improved survival in high-risk patients [62]. Elevated IL-10 has been consistently associated with adverse outcomes and is increasingly regarded as a marker for intensified monitoring and timely supportive care [70]. In contrast, although S100B shows a strong association with pneumonia and disease progression, its role in guiding early intervention strategies remains insufficiently defined [56]. Collectively, these findings indicate that while these biomarkers hold promise for early risk stratification, further prospective studies are needed to validate their utility in improving patient outcomes.

However, further research is needed to standardize the measurement of these biomarkers across different clinical settings. More rigorous and consistent protocols will enable better comparability of future studies and enhance the clinical applicability of these biomarkers. Additionally, longitudinal studies assessing the dynamics of these biomarkers over time will provide insights into their role in predicting long-term outcomes, such as the post-acute sequelae of COVID-19. As research advances, combining multi-omics approaches, such as proteomics, genomics, and metabolomics, with biomarker research could reveal new molecular pathways and therapeutic targets for managing severe COVID-19. Furthermore, comparative analyses between COVID-19 biomarkers and those of other respiratory infections could help identify specific disease markers and facilitate the development of targeted therapeutic strategies.

### Limitations and Strength

The strengths of this study encompass a thorough analysis that consolidates data from numerous research efforts, offering a wide-ranging assessment of S100 proteins and interleukins (IL-6, IL-10) across different stages of COVID-19 severity, thereby delivering important insights into their clinical significance. Moreover, the study reveals consistent results for crucial biomarkers, such as IL-6 and S100B, exhibiting low variability (I^2^ < 30%) among the studies, which suggests dependable associations with COVID-19 severity, particularly in pneumonia cases. On the downside, the study also faces limitations. Incomplete reporting of demographic variables such as age, sex distribution, and BMI across the included studies limited our ability to assess their influence on biomarker levels. The examination of IL-10 levels demonstrated significant variability (I^2^ = 90%), likely attributed to differences in study methodologies, patient demographics, or the timing of cytokine assessments, which impacts the reliability of these findings. Additionally, certain analyses, especially those regarding S100 proteins, relied on a limited number of studies, potentially diminishing the generalizability of the results and highlighting the need for further investigation.

### Future Directions

Future research should focus on longitudinal studies that monitor S100 proteins and interleukins to gain deeper insights into their roles in COVID-19 progression and recovery. Including more diverse populations in these studies would help identify biomarker variability, enhancing the relevance of findings across different demographic and ethnic groups. Investigating the potential of IL-6, IL-10, and S100 proteins as therapeutic targets may pave the way for personalized treatments aimed at mitigating inflammation and reducing severe outcomes. Combining multi-omics approaches, such as proteomics, genomics, and metabolomics, with biomarker research could reveal new molecular pathways and targets in severe COVID-19 cases. Additionally, comparative analyses between COVID-19 biomarkers and those of other respiratory infections could help identify specific disease markers and facilitate the development of targeted therapeutic strategies.

## Conclusion

This meta-analysis provides robust evidence that inflammatory biomarkers, particularly IL-6, IL-10, IL-8, and S100B, are significantly elevated in patients with COVID-19 pneumonia and organ failure compared to non-pneumonia COVID-19 patients and healthy controls. These markers reflect the immune dysregulation and hyperinflammatory states that exists in severe COVID-19, confirming IL-6’s central role and extending attention to IL-10, IL-8, and S100B as complementary markers of disease severity. Together, they delineate a broader biomarker signature that has potential value for early risk stratification, prognosis, and therapeutic targeting. Looking at these markers together gives a fuller picture of both inflammation and tissue damage. While IL-6 inhibition is already clinically validated, the roles of IL-10, IL-8, and S100B suggest additional pathways and markers that could be integrated into clinical practice for monitoring and decision-making. More research is needed to standardize how these markers are measured, test how well they predict outcomes in different patient groups and see how they can be used in practice. Studying these markers further will not only improve care but also deepen our understanding of severe outcomes and inflammatory cascades in general.

## Summary Table

### What Is Known About This Subject

- COVID-19 severity ranges from mild infection to pneumonia, Acute Respiratory Distress Syndrome (ARDS), and organ failure (=111).
- Interleukins such as IL-6 and IL-10 are linked to inflammation and disease severity (=92).
•S100 proteins contribute to inflammation and indicate progression of COVID-19 (=90).

### What This Paper Adds

- IL-6 is significantly elevated in COVID-19 patients with pneumonia versus those without (=94).
- IL-10 is elevated in pneumonia cases, supporting its role in severe disease response (=91).
- S100B shows significant association with pneumonia in COVID-19 patients (=85).

### Concluding Statement

This work represents an advance in biomedical science because it consolidates evidence that IL-6, IL-10, and S100B are reliable biomarkers for assessing COVID-19 pneumonia severity and progression (=194).

## Data Availability

The original contributions presented in the study are included in the article/supplementary material, further inquiries can be directed to the corresponding authors.
