# Supplementary material for: S100 Protein and Interleukin Biomarkers Among COVID-19 Subjects With and Without Pneumonia: A Systematic Review and Meta-Analysis
Source: Br J Biomed Sci. 2026 Jan 8;82:15355. doi: 10.3389/bjbs.2025.15355 (PMC12823546; doi:10.3389/bjbs.2025.15355)
Supplement: Supplementary file 1 [file Table1.pdf]

Appendix 1 – Search Strategy

PubMed

	Search terms	Hits
#1	"SARS-CoV-2"[Mesh] OR "COVID-19"[Mesh] OR "SARS-CoV"[tiab] OR "SARS CoV"[tiab] OR "novel coronavirus"[tiab] OR "nCoV"[tiab] OR "2019-nCoV"[tiab] OR COVID[tiab] OR "severe acute respiratory syndrome coronavirus"[tiab] OR "Coronavirus Disease 2019"[tiab]	436,650
#2	"S100 Proteins"[Mesh] OR "S100A4 protein, human"[tiab] OR "S100 Protein"[tiab] OR "S100A4"[tiab] OR "S100A4 protein, human"[tiab] OR "S100A4 protein" [tiab] OR "S100A8"[tiab] OR "S100A8 protein" [tiab] OR "S100A9"[tiab] OR "S100A9 protein" [tiab] OR "S100A12"[tiab] OR "S100A12 protein" [tiab] OR "S100B"[tiab] OR "S100B protein, human"[tiab] OR "S100B protein" [tiab] OR "S100P"[tiab] OR " S100 calcium binding protein P" [tiab] OR Interleukins [Mesh] OR "Interleukin 6" [tiab] OR "IL-6"[tiab]OR IL6 [tiab] OR "Interleukin 8" [tiab] OR "IL-8" [tiab] OR IL8[tiab] OR "IL-10" [tiab] OR IL10[tiab] OR "Interleukin 10" [tiab] OR "IL-17" [tiab] OR "Interleukin 17" [tiab] OR "Interleukin 1beta" [tiab] OR "IL-1 beta" [tiab] OR "Interleukin-1 beta" [tiab] OR"Interleukin 1 beta" [tiab] OR"IL-1β" [tiab]	501,996
#3	biomarker*[MeSH Terms] OR “biological marker*”[MeSH Terms] OR "Prevalence"[Mesh] OR occurrence[tiab] OR frequency[tiab] OR expression[tiab] OR SNP[tiab]OR "Genetic Polymorphism"[tiab]	4,943,627
#4	Pneumonia[Mesh] OR "Multiple Organ Failure"[Mesh] OR "Organ failure"[tiab] OR "Organ failures"[tiab] OR "Organ dysfunction"[tiab] OR "Organ dysfunctions"[tiab] OR "Organ system dysfunction"[tiab] OR "Organ system dysfunctions"[tiab] OR "Multiple organ failure"[tiab] OR pneumonia[tiab] OR pneumonias[tiab]	510,964
	#1 AND #2 AND #3 AND #4	1803
	#1 AND #2 AND #3 AND #4 Filters: Humans, from 2020 - 2024	**1702**

Web of Science

	Search terms	Hits
#1	(TI=("SARS-CoV-2" OR "COVID-19" OR "SARS-CoV" OR "novel coronavirus" OR "nCoV" OR "2019-nCoV" OR COVID OR "severe acute respiratory syndrome coronavirus" OR "Coronavirus Disease 2019")) OR AB=("SARS-CoV-2" OR "COVID-19" OR "SARS-CoV" OR "novel coronavirus" OR "nCoV" OR "2019-nCoV" OR COVID OR "severe acute respiratory syndrome coronavirus" OR "Coronavirus Disease 2019")	556,066
#2	(AB=(S100 Protein*” OR "S100A4" OR "S100A4 protein" OR "S100A8" OR "S100A8 protein" OR "S100A9" OR "S100A9 protein" OR "S100A12" OR "S100A12 protein" OR S100B OR "S100B protein" OR S100P OR "S100 calcium binding protein P" OR Interleukins OR "Interleukin 6" OR "IL-6" OR IL6 OR Interleukin 8" OR "IL-8" OR IL8 OR "IL-10" OR IL10 OR "Interleukin 10" OR "IL-17" OR "Interleukin 17" OR "Interleukin 1beta" OR "IL-1 beta" OR "Interleukin-1 beta" OR "Interleukin 1 beta" OR"IL-1β")) OR TI=(S100 Protein*” OR "S100A4" OR "S100A4 protein" OR "S100A8" OR "S100A8 protein" OR "S100A9" OR "S100A9 protein" OR "S100A12" OR "S100A12 protein" OR S100B OR "S100B protein" OR S100P OR "S100 calcium binding protein P" OR Interleukins OR "Interleukin 6" OR "IL-6" OR IL6 OR Interleukin 8" OR "IL-8" OR IL8 OR "IL-10" OR IL10 OR "Interleukin 10" OR "IL-17" OR "Interleukin 17" OR "Interleukin 1beta" OR "IL-1 beta" OR "Interleukin-1 beta" OR "Interleukin 1 beta" OR"IL-1β")	244,644
#3	TI=(“biomarker*” OR “biological marker*” OR Prevalence OR occurrence OR frequency OR expression OR SNP OR "Genetic Polymorphism")) OR AB=(“biomarker*” OR “biological	7,717,847

	marker*" OR Prevalence OR occurrence OR frequency OR expression OR SNP OR "Genetic Polymorphism")	
#4	(TI=(Pneumonia OR "Multiple Organ Failure" OR "Organ failure" OR "Organ failures" OR "Organ dysfunction" OR "Organ dysfunctions" OR "Organ system dysfunction"[OR "Organ system dysfunctions" OR "Multiple organ failure" OR pneumonia OR pneumonias)) OR AB=(Pneumonia OR "Multiple Organ Failure" OR "Organ failure" OR "Organ failures" OR "Organ dysfunction" OR "Organ dysfunctions" OR "Organ system dysfunction"[OR "Organ system dysfunctions" OR "Multiple organ failure" OR pneumonia OR pneumonias)	182,998
	#1 AND #2 AND #3 AND #4	**114**

Scopus

	**Search terms**	**Hits**
	(TITLE-ABS-KEY("SARS-CoV-2" OR "COVID-19" OR "SARS-CoV" OR "novel coronavirus" OR "nCoV" OR "2019-nCoV" OR COVID OR "severe acute respiratory syndrome coronavirus" OR "Coronavirus Disease 2019")) AND ("S100 Protein*" OR "S100A4" OR "S100A4 protein" OR "S100A8" OR "S100A8 protein" OR "S100A9" OR "S100A9 protein" OR "S100A12" OR "S100A12 protein" OR S100B OR "S100B protein" OR S100P OR "S100 calcium binding protein P" OR Interleukins OR "Interleukin 6" OR "IL-6" OR IL6 OR "Interleukin 8" OR "IL-8" OR IL8 OR "IL-10" OR IL10 OR "Interleukin 10" OR "IL-17" OR "Interleukin 17" OR "Interleukin 1beta" OR "IL-1 beta" OR "Interleukin-1 beta" OR "Interleukin 1 beta" OR"IL-1β") AND (TITLE-ABS-KEY("biomarker*" OR "biological marker*" OR Prevalence OR occurrence OR frequency OR expression OR SNP OR "Genetic Polymorphism")) AND (TITLE-ABS-KEY(Pneumonia OR "Multiple Organ Failure" OR "Organ failure" OR "Organ failures" OR "Organ dysfunction" OR "Organ dysfunctions" OR "Organ system dysfunction"[OR "Organ system dysfunctions" OR "Multiple organ failure" OR pneumonia OR pneumonias)) AND PUBYEAR > 2019 AND PUBYEAR < 2025 AND (LIMIT-TO (DOCTYPE,"ar")) AND (LIMIT-TO (LANGUAGE,"English"))	**2,045**

Cochrane Library/CENTRAL

	**Search terms**	**Hits**
#1	("SARS-CoV-2" OR "COVID-19" OR "SARS-CoV" OR "novel coronavirus" OR "nCoV" OR "2019-nCoV" OR COVID OR "severe acute respiratory syndrome coronavirus" OR "Coronavirus Disease 2019"):ti,ab,kw	21714
#2	MeSH descriptor: [Coronavirus] this term only	17
#3	MeSH descriptor: [COVID-19] explode all trees	7987
#4	#1 OR #2 OR #3	21725
#5	MeSH descriptor: [S100 Proteins] explode all trees	568
#6	(S100A4 OR "S100A4 protein" OR S100A8 OR "S100A8 protein"):ti,ab,kw	104
#7	(S100A9 OR "S100A9 protein" OR S100A12 OR "S100A12 protein" OR S100B OR "S100B protein" OR S100P OR "S100 calcium binding protein P"):ti,ab,kw	496
#8	(Interleukins OR "Interleukin 6" OR "IL-6" OR IL6):ti,ab,kw	23317
#9	(Interleukin 8 OR IL-8 OR IL8 OR "IL-10" OR IL10):ti,ab,kw	16165
#10	#5 OR #6 OR #7 OR #8 OR #9	30771
#11	#4 AND #10	1108
#12	(biomarker OR prevalence OR SNP OR "Genetic Polymorphism"):ti,ab,kw	67467
#13	#11 AND #12	53
#14	MeSH descriptor: [Multiple Organ Failure] explode all trees	560
#15	MeSH descriptor: [Pneumonia] explode all trees	12925
#16	("Multiple Organ Failure" OR "Organ failure" OR "Organ failures" OR "Organ dysfunction" OR "Organ dysfunctions" OR "Organ system dysfunction" OR "Organ system dysfunctions" OR "Multiple organ failure" OR pneumonia OR pneumonias):ti,ab,kw	27751

#17	#14 OR #15 OR #16	34720
#18	#13 AND #17	**32**
